# Termite Societies Promote the Taxonomic and Functional Diversity of Archaeal Communities in Mound Soils

**DOI:** 10.3390/biology9060136

**Published:** 2020-06-25

**Authors:** Monde Wakung’oli, Adenike Eunice Amoo, Ben Jesuorsemwen Enagbonma, Olubukola Oluranti Babalola

**Affiliations:** Food Security and Safety Niche, Faculty of Natural and Agricultural Sciences, North-West University, Private Mail Bag X2046, Mmabatho 2735, South Africa; mwakungoli@yahoo.com (M.W.); adeyemoadenike11@yahoo.com (A.E.A.); benjamin.enagbonma@uniben.edu (B.J.E.)

**Keywords:** bioturbators, Illumina MiSeq, shotgun sequencing, soil physicochemical properties, South Africa, termitarium

## Abstract

Recent studies involving microbial communities in termite mounds have been more focused on bacteria and fungi with little attention given to archaea, which play significant roles in nutrient cycling. Thus, we aimed at characterizing the archaeal taxonomic and functional diversity in two termite mound soils using the shotgun sequencing method with the assumption that termite activities could promote archaeal diversity. Our findings showed that termite mound soils have archaeal groups that are taxonomically different from their surrounding soils, with *Euryarchaeota*, *Korarchaeota*, and *Nanoarchaeota* being predominant while *Thaumarchaeota* and *Crenarchaeota* were predominant in the surrounding soils. Additionally, the observed nutrient pathways: phosphorus, nitrogen, and sulfur were all significantly more predominant in termite mound soils than in their comparative surrounding soils. Alpha diversity showed that archaea were not significantly different within termite mound soils and the surrounding soils. The beta diversity revealed significant differences in the archaeal taxonomic composition and their functional categories between the termite mounds and surrounding soils. Our canonical correspondence analysis revealed that the distribution of archaeal communities was likely dependent on the soil properties. Our results suggested that termite activities may promote the diversity of archaea; with some of our sequences grouped as unclassified archaea, there is a need for further research to unveil their identity.

## 1. Introduction

Mound-building termites from an agricultural perspective are seen as pests, since they affect crops and farm structures, therefore leading to profit loss [1]. Nonetheless, termites, due to their mound-building activities, have shown positive effects in the soils they inhabit [2,3]. Termites are one of the most crucial soil bioturbators [4]; they excavate organic materials and mineral particles from different depths of their parent soil, which they mix with their saliva and excretions to form building materials when constructing their mounds [5]. Several reviews have shown that termite activities during mound construction influence the physicochemical properties of mound soils [4,6,7]. This makes termite mound soils richer in organic carbon, clay, silt, magnesium, phosphorus, potassium, and calcium than their surrounding soils [4,8,9]. Since the activities of termites promote the physical and chemical properties of soils, soil fertility is consequently enhanced, thereby making the soil refugia for microbial concentrations [3,10]. 

Current studies have revealed that termite societies can increase the activity of soil bacterial [11] and fungal [12] communities and change their community composition at the scale of individual mounds. Little is known, however, about archaeal communities and their functional abilities in termite mound soils. Profiling the archaeal taxonomic and functional diversity in termite mound soils is vital for understanding and manipulating ecosystems for industrial or research purposes. Thus, this study aimed at characterizing archaeal structural and functional diversity in termite mounds soils. 

Many studies that have been carried out so far on termite mound soil microorganisms are based on culture-dependent and sanger-sequencing methods. These methods only offer little data about microbial diversity [13]. However, Manjula et al. [14] and Makonde et al. [11] recently used amplicon sequencing methods to explore the structure of bacterial communities in termite mounds. They assessed certain regions of the bacterial DNA. From their conclusions, most of the reads were grouped up to the domain level, and they never investigated the functional ability of the bacteria. To classify the bulk of our reads past the level of the domain and evade the shortcomings related to amplicon sequencing [15,16], we employed the shotgun metagenomic sequencing method. This enabled us to comprehensively profile the archaeal structural and functional diversity in termite mound soils relative to their adjacent soils. This method was used to answer two basic questions: (1) Are the taxonomic and functional diversity of archaeal communities in termite mound soils significantly different from their surrounding soils? (2) Which soil properties best influence the archaeal community distribution? This study, to the best of our awareness, is the first to examine archaeal communities in termite mound soils using the shotgun metagenomic method.

## 2. Materials and Methods

### 2.1. Study Site and Sample Collection

We collected sixteen (16) soil samples from termite mounds and their surrounding soils in August 2017. That is four (4) soil samples from termite mounds (T1) and four soil samples from their comparative surrounding soils at Braklaagte (S1). We also collected four (4) soil samples from termite mound soils (T2) and four (4) soil samples from their comparative surrounding soils at Zeerust (S2). Braklaagte (25°26′13.5″ S 26°05′50.4″ E) and Zeerust (25°27′11.2″ S 26°07′33.8″ E) are located in North West Province, South Africa. The mean air temperature of the area ranges from 3 °C to 21 °C and 17 °C to 31 °C in winter and summer, respectively. The annual precipitation of the province is roughly 360 mm, with most falling in the middle of October and April [17]. A 5 cm diameter split tube auger was used to collect soil samples of 50 g at a 1 m depth from termite mound soils populated by the *Coptotermes* species. The distance between the termite mounds and their comparative surrounding soils in each location was 10 m. The soil samples were preserved in cooler boxes filled with ice blocks during the sampling, transported to the Microbial Biotechnology Laboratory under frozen conditions, and stored until analysis. The soil samples were divided into two parts: (1) for physical-chemical analysis (stored in a fridge at 4 °C) and (2) for DNA analysis (stored in a freezer at −80 °C) for 14 days. After the soil analysis, the mean values were used for a statistical analysis.

### 2.2. Soil Physical and Chemical Parameters

The soil’s physical and chemical characteristics were analyzed using standard procedures. Soil particle size analyses were performed using the hydrometer method [9]. The United States Department of Agriculture (USDA) particle size classes (clay (<0.002 mm); silt (0.05–0.002 mm); and sand (2.0–0.05 mm)) were followed for assigning textural classes. The soil pH in water was evaluated using a pH meter in the ratio of 1:2.5 (soil: water); the phosphorus (P) and potassium ions (K^+^) were analyzed by colorimetry and flame photometry, respectively [18]. The total nitrogen (N) was determined by the Kjeldhal method. Calcium ions (Ca^+2^) and magnesium ions (Mg^+2^) were extracted using the 1 M ammonium acetate method at pH 7.0 and evaluated using an atomic absorption spectrophotometer. The determination of the organic carbon content was carried out using the dichromate digestion [19,20].

### 2.3. DNA Extraction

The metagenomic DNA was extracted from 0.25 g of each sample collected from termite mound soils and their corresponding surrounding soils using the PowerSoil^®^ DNA isolation kit (MoBio Laboratories Inc., Carlsbad, CA, USA) following the manufacturer’s instructions. The concentration of the extracted DNA was measured by fluorescence using the Quant-iT PicoGreen dsDNA kit (Invitrogen, Carlsbad, CA, USA), which was assessed on a DQ 300 fluorometer (Hoefer Scientific Instruments, San Francisco, CA, USA). The extracted DNA was stored at −80 °C while awaiting sequencing.

### 2.4. Metagenomic DNA Sequencing 

We used the Illumina MiSeq 2500 platforms (San Diego, CA, USA) for sequencing the extracted DNA. All the datasets were created by whole metagenome shotgun sequencing at Molecular Research LP (MR DNA, Shallowater, TX, USA). An amount of 50 ng of DNA from each sample was used to construct library sequencing via the Nextera DNA Sample Preparation Kit (Illumina). The library insert size was assessed with an Experion Automated Electrophoresis Station (Bio-Rad). The insert size of the libraries alternated from 300 to 850 bp (average 500 bp). Each library was loaded to a 600 Cycles v3 Reagent cartridge (Illumina), and the sequencing was performed using a 2 × 250 base pair sequencing run. 

### 2.5. Metagenomic Data Analysis

The raw sequences of each of the metagenomes were uploaded to the metagenomics rapid annotation online server (MG-RAST) at https://www.mg-rast.org (accessed on 10 July 2019) [21]. In the MG-RAST server, the sequences were subjected to quality control. This comprised of dereplication—that is, the removal of artificial sequences formed by sequencing artifacts, removing host-specific species sequences, ambiguous base filtering (removing sequences with >5 ambiguous base pairs with a 15 phred score cutoff) and length filtering (removing sequences with a length of >2 standard deviation from the mean). Following quality control (QC), the sequences were annotated using the BLAT (the BLAST-like alignment tool) algorithm [22] against the M5NR database [23], which provides a nonredundant integration of many databases. Archaeal classifications were performed by the SEED subsystem. An e-value of 1e−5, a minimum identity of 60%, and a maximum alignment length of 15 base pairs were the conditions used when the archaeal classifications were assigned. No further analyses were carried out on sequences that failed annotation. Our focus was on archaea; hence, we discarded sequences obtained from viruses, bacteria, and eukaryotes. To decrease the effect of experimental noise/error, the normalized data option of MG-RAST was applied. Functional classification was performed using the Systems Biology Knowledgebase (KBase) (https://kbase.us/) (accessed on the 23rd of September, 2019), which is a comprehensive stand-alone framework. The genomes were annotated using the Prokka annotation pipeline and non-redundant (NR) database. The predicted genes were used to hit the Kyoto Encyclopedia of Genes and Genomes (KEGG) and the non-redundant protein (NR) database via the KBase pipeline. The resulting archaeal table was agglomerated accordingly to each taxa level, and unclassified reads were retained for statistical purposes. Next, the abundances (Supplementary Tables S2–S5) were transformed into percentages. The mean values of the relative abundances of all 4 samples from each site (T1, T2, S1, and S2) were used for statistical analysis. The quality sequences are available from the Sequence Read Archive (SRA) of the National Center for Biotechnology Information (NCBI)dataset under the bioproject numbers PRJNA526912 (for termite mound soil samples) and PRJNA525146 (for the surrounding soil samples).

### 2.6. Statistical Analysis

The differences between physicochemical parameters were determined by a one-way analysis of variance (ANOVA) for the comparison of means with Tukey’s pairwise comparison test for significance level (*p* < 0.05). Pielou Evenness and Shannon diversity indices were assessed for each of the samples and these indices were compared between habitats using a Kruskal–Wallis test. All these analyses were performed using PAST version 3.20 [24]. The beta diversity was depicted using a principal coordinate analysis (PCoA) based on an Euclidean distance matrix, and a one-way analysis of similarities (ANOSIM) via 999 permutations was used to test for differences in community composition between the groups of samples [25]. A principal component analysis (PCA) based on a Euclidean distance matrix was used to show how these archaeal communities were distributed between the termite mound and the surrounding soil samples. To find the environmental variables that best explained the archaeal composition, we performed a canonical correspondence analysis (CCA) and applied a forward selection of environmental variables; the Monte Carlo permutation test with 999 random permutations was used for a significance test. All the environmental variables listed in Table 1 were included in the CCA analysis as explanatory variables. The PCoA and PCA plots were generated using PAST version 3.20, while the CCA was plotted using CANOCO 5 (Microcomputer Power, Ithaca, NY, USA). The Heatmap was drawn using the Shinyheatmap, with a z-score-transformed relative abundance of archaeal gene categories [26]. 

## 3. Results 

### 3.1. Physical and Chemical Characterization of Termite Mound Soils and Their Surrounding Soils

The soil analysis showed that clay and K were significantly higher in termite mound soils in relation to the surrounding soils. Meanwhile, sand, pH, and N were significantly higher in the surrounding soils compared to the termite mounds (Table 1). The individual values before the means were collated are found in Supplementary Table S1.

### 3.2. Sequencing Data Analyses of the Samples Collected from Termite Mounds and the Surrounding Soils

The average numbers of uploaded sequences were 6,984,205 (T1) and 6,949,161 (T2) sequence reads for the termite mound soil samples and 7,490,918 (S1) and 7,057,133 (S2) sequence reads for the surrounding soil samples. After quality control (QC) was executed in MG-RAST [21], the quantities of retained average sequences for the termite mound soil samples were 6,802,220 (T1) and 6,422,685 (T2), with an average G + C content of 61.25%, while the sequences of the surrounding soil samples were 7,327,766 (S1) and 6,916,304 (S2), with an average G + C content of 66.25%. In the termite mounds from Braklaagte (T1), it was observed that the sequences of archaeal origin on average made up 0.77% of the entire metagenome. Bacterial communities made up 97.79% of the sequences, eukaryotes comprised 1.3%, and viruses 0.02%, while 0.12% of the sequences were unclassified. Termite mounds from Zeerust (T2) had an average of 0.43% archaeal sequences, 92.60% bacterial sequences, 6.80% sequences from eukaryotes, 0.09% of viral origin, and 0.08% unclassified sequences. The surrounding soils from Braklaagte (S1) had 0.65% sequences of archaeal origin, 98.60% bacterial sequences, 0.65% sequences from eukaryotes, and 0.02% viral sequences, while 0.09% were unclassified. The surrounding soils from Zeerust (S2) were made up of 0.59% archaeal sequences, 98.60% bacterial sequences, 0.71% eukaryotic sequences, 0.01% sequences from viruses, and 0.09% unclassified sequences.

### 3.3. Structural Composition Analysis of the Archaeal Community

**Figure 1:**
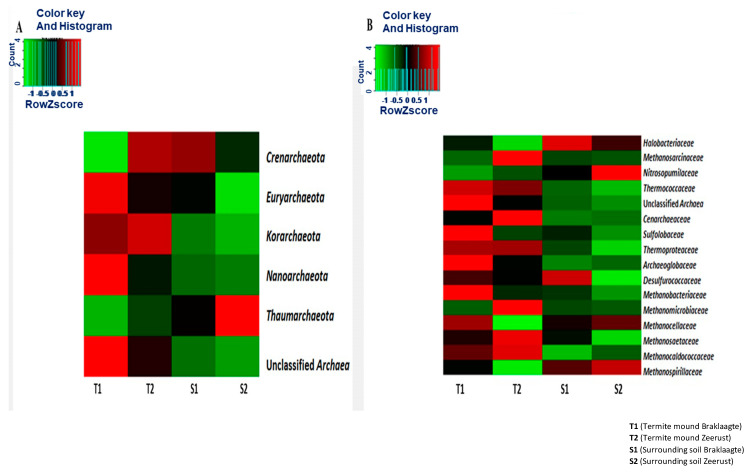
Relative abundance of the archaeal communities at the (**A**) phylum level and (**B**) family level across sites. The scale bar shows the color saturation gradient based on the relative abundances, with a z-score-transformed relative abundance of archaeal communities.

At the phylum level (Figure 1a), *Euryarchaeota*, *Korarchaeota,* and *Nanoarchaeota* were predominant (*p*-value < 0.05) in the termite mounds, while *Crenarchaeota* and *Thaumarchaeota* predominated in the surrounding soils (Figure 1a). At the family level (Figure 1b), *Thermococcaceae, Cenarchaeoceae*, *Thermoproteaceae*, *Archaeoglobaceae*, *Desulfurococcaceae Methanosaetaceae*, and *Methanocaldococcaceae* were more abundant in the termite mounds, while *Halobacteriaceae*, *Nitrosopumilaceae*, *Methanocelloceae*, and *Methanospirillaceae* were more abundant in the surrounding soils (Figure 1b). A principal component analysis (PCA) was used to show the archaeal distributions at the genus level (Supplementary Figure S1). It showed that at the genus level, *Cenarchaeum*, *Thermococcus*, and *Sulfolobus* were abundant in the termite mound soils, while *Nitrosopumilus*, *Candidatus Nitrososphaera*, *Haloterrigena*, *Haladaptatus*, *Methanocella*, and *Methanoculleus* were more abundant in the surrounding soils.

### 3.4. General Metabolism Categories

**Figure 2:**
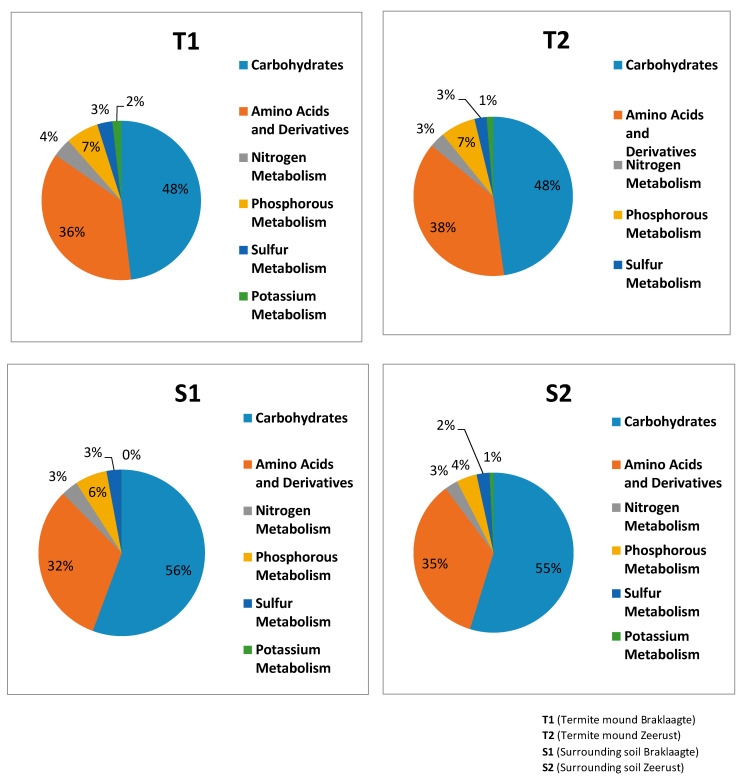
The relative abundance of the major functional categories in each soil sample.

**Figure 3:**
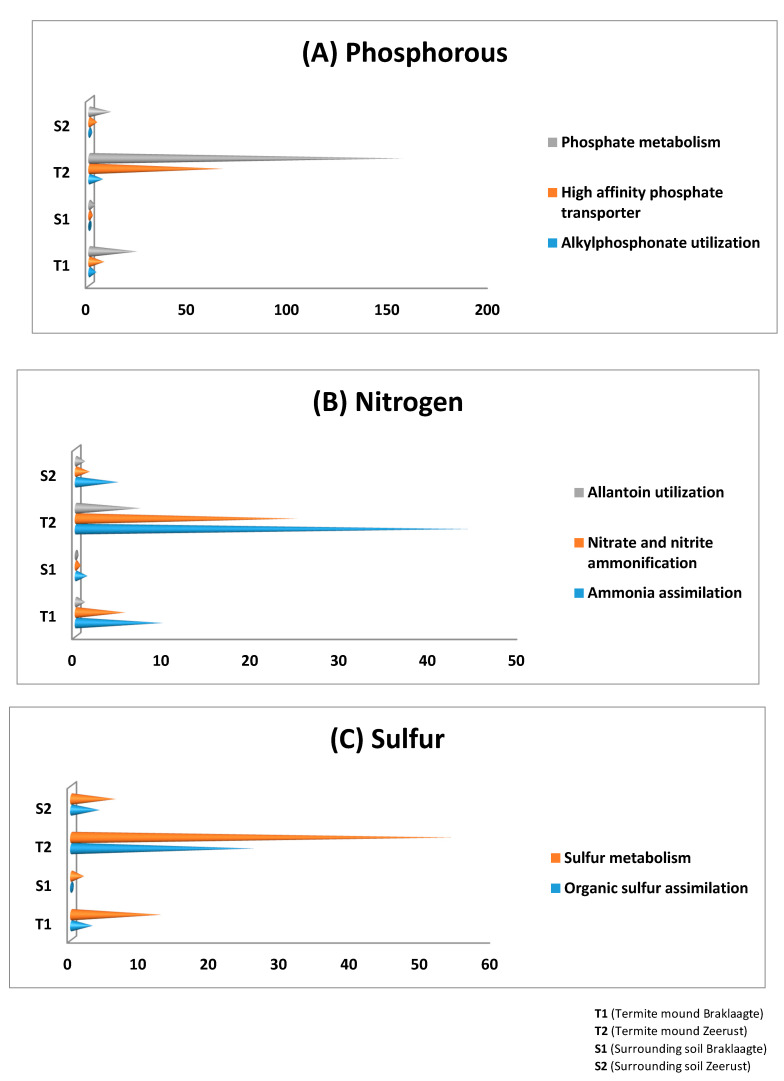
The relative abundance of the (**A**) phosphorus, (**B**) nitrogen, and (**C**) sulfur pathways obtained from termite mound soils and their surrounding soil samples.

It can be observed from the results that amino acids and derivatives and carbohydrates metabolism dominated both the termite mounds and the surrounding soils (Figure 2). The nutrient pathways phosphorus, nitrogen, and sulfur were all significantly (*p*-values < 0.05) more predominant in termite mound soils than their comparative surrounding soils (Figure 3).

### 3.5. Alpha and Beta Diversity Estimations for Archaeal Communities from Termite Mounds and Surrounding Soils

**Figure 4:**
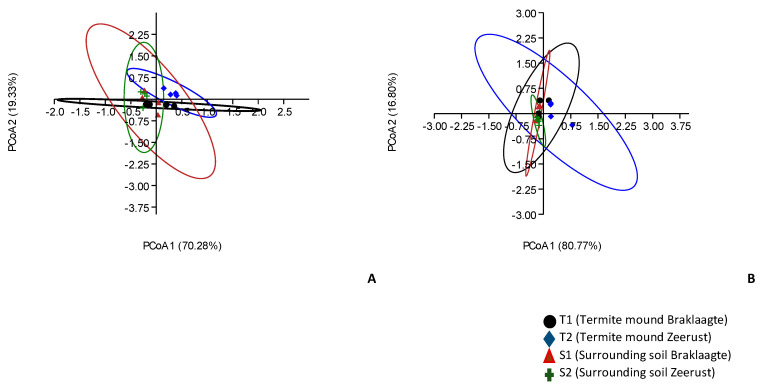
Principal coordinates analysis (PCoA) of (**A**) archaeal taxonomic composition and (**B**) functional categories of termite mound soils and their surrounding soils.

Shannon and Evenness indices were used to depict the alpha diversity (that is, diversity within habitats) of the archaeal community. The Shannon and Evenness indices showed that there were no significant differences in the alpha diversity of the archaeal taxonomic composition (Kruskal–Wallis, *p*-value = 0.99) and their functional categories (Kruskal–Wallis, *p*-value = 0.20) (Table 2). However, there was a significant difference in the beta diversity (that is diversity between habitats) for the archaeal taxonomic composition (ANOSIM, *p*-values = 0.03; R = 0.17), while the functional categories were not significantly different (ANOSIM, *p*-values = 0.82; R = −0.09)), as depicted with the PCoA (Figure 4).

### 3.6. Influence of Environmental Factors on Archaeal Community

**Figure 5:**
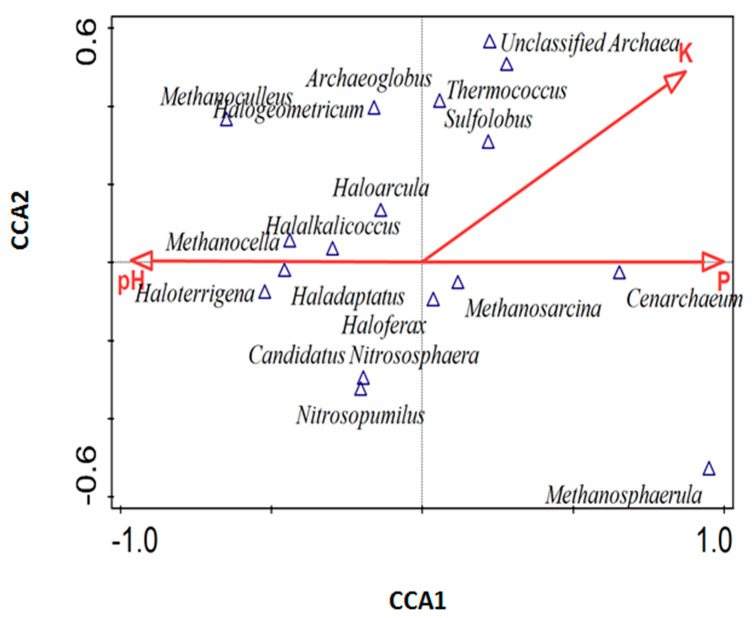
Canonical correspondence analysis (CCA) of the archaeal community distribution and soil physicochemical properties of both termite mound soils and the surrounding soil samples.

To determine the effect of soil properties on the archaeal community distribution, a canonical correspondence analysis (CCA) was used (Figure 5). All the soil physical and chemical properties in Table 1 were used for the CCA plot (Figure 5). The canonical correspondence analysis (CCA) plot (Figure 5) indicated that the composition of the archaeal communities was likely dependent on the soil properties. The vector length of K (on the axis 1) positively correlated with *Thermococcus* and *Sulfolobus*. On axis 2, the vector length of P positively correlated with *Cenarchaeum*, *Methanosarcina*, and *Methanosphaerula*, but negatively correlated with *Haloterrigena*.

## 4. Discussion

In this research, we investigated and characterized the archaeal communities in termite mound soils relative to their surrounding soils using the shotgun metagenomics sequencing approach. Shotgun metagenomics has shown its importance and usefulness in evaluating the composition and abundance of microbial communities [19,27]. This method can profile archaeal functional capabilities unlike the use of amplicon sequencing method, which only profiles archaeal species [28,29].

Firstly, to answer the question “are the taxonomic and functional diversity of archaeal communities in termite mound soils significantly different from their surrounding soils?”, Shannon and Evenness indices were employed to analyze the alpha diversity. The alpha diversity showed that archaea were not significantly different (*p*-value > 0.05) between termite mound soils and the surrounding soils (Table 2). The alpha diversity also indicated that only the functional diversity represented by the metagenomes in termite mound soils passed its hypothetical limit of 2.81 [30], signifying that most metagenomes were characterized in the termite mound soils samples. The Evenness indices for the metagenomes across all samples were low (<1, Table 2), signifying that there are a few dominant taxa (like *Euryarchaeota*, *Korarchaeota*, and *Nanoarchaeota*) and functional categories (like carbohydrate and amino acids and derivatives) in each environment. Furthermore, we used the PCoA plot to depict the beta diversity, which is the diversity between the termite mound soils and their surrounding soils. The PCoA plot revealed that termite mound soil samples (T1 and T2) formed no distinct clustering with the surrounding soil samples (S1 and S2) (Figure 4a). This means there was a significant difference (*p*-value < 0.05) in the archaeal community taxonomic composition between the termite mounds and the surrounding soils, as shown by the analysis of similarity (ANOSIM), and the strength of the difference was R = 0.17. There was no significant difference in the functional categories that were considered between the mound soils and the surrounding soils. Differences in the bacterial community structure have been described by various studies [11,12]. Termites have been reported to impact soil chemical parameters, and this consequently enables them to influence the diversity of other organisms inhabiting the soil. Due to their building actions and mounds, termites have effects on soil microbial communities [12].

To answer if each environment had distinguished archaeal communities, a principal component analysis (PCA) was plotted. Our PCA analysis showed, for example, that *Cenarchaeum*, *Methanosphaerula*, and *Methanosarcina* predominated the termite mound soil (T2), while *Candidatus Nitrososphaera*, *Nitrosopumilus*, and *Haloferax* predominated the surrounding soil (S2) (Figure S1). The discrepancies in the archaeal domination in both habitats might also influence the ecological roles played by these archaea. Archaea have the ability to recycle soil nutrients like nitrogen in environments [31]. Furthermore, methane-oxidizing archaea like *Methanosarcina* (which was predominant in T2) can act as biofilter [32,33] and reduce the amount of methane finally released to the surroundings [32]. This could be the reason why the phosphorus (phosphate metabolism, alkylphosphonate, and high-affinity phosphate transporter), nitrogen (ammonia assimilation, nitrate and nitrite ammonification, and allantoin utilization), and sulfur (organic sulfur assimilation and sulfur metabolism) pathways were all significantly more predominant in termite mound soils than in their comparative surrounding soils (Figure 3).

The differences in the archaeal domination in each habitat could be as a result of the physicochemical differences that existed between the termite mounds and the surrounding soil samples (Table 1). Our physicochemical analysis showed that the pH was meaningfully (*p* < 0.05) lower in termite mound soils than the surrounding soils (Table 1). Zheng et al. [34] reported that pH is a strong soil factor that influences microbial diversity. Furthermore, termite mound soils were also significantly richer in clay and K in relation to the surrounding soils. Although the values of P, Mg, and Ca were also higher in termite mound soils, they did not differ meaningfully from the surrounding soil samples. The canonical correspondence analysis (CCA) plot showed (Figure 5) that P alone explained 55.3% of the entire variation in the archaeal communities. Considering the vector length of P and K in our CCA plot, it showed that not only pH could determine the shaping of the archaeal communities. Faoro et al. [35] and Dhembare (2013) demonstrated the importance of Ca, Mg, P, and OC contents in shaping the soil microbial community composition.

This study also revealed many unclassified groups of archaea, and this could point to the presence of potentially novel species, as they could not be classified into the current species (Probst and Moissl-Eichinger 2015; Liu et al., 2018). Making out plans to isolate and culture these archaea could give room for the discovery of novel archaeal species for industrial uses.

## 5. Conclusions

This study, to the best of our knowledge, is the first of its kind to profile archaeal diversity in termite mound soils using the shotgun sequencing approach. The alpha diversity showed that archaea were not significantly different within termite mound soils and the surrounding soils. Furthermore, the beta diversity showed significant differences in the archaeal taxonomic composition, although the functional categories between the termite mounds and surrounding soils were not significantly different. We also reported that *Euryarchaeota*, *Korarchaeota*, and *Nanoarchaeota* were more predominant in termite mound soils, while *Thaumarchaeota* and *Crenarchaeota* were more predominant in the surrounding soils. Additionally, all observed nutrient pathways were predominant in termite mound soils in contrast to their surrounding soils. Furthermore, our canonical correspondence analysis revealed that soil properties influenced the archaeal distribution, with P explaining most of the entire variation. The presence of sequences assigned to unclassified archaea in this study could suggest the need for further research to unveil their identity.

## Figures and Tables

**Table 1:** Soil properties in termite mound soils and surrounding soils.

Soil Property	T1	T2	S1	S2
pH	5.10 ± 0.33 a	4.48 ± 0.46 a	5.80 ± 0.32 b	5.38 ± 0.39 c
N (%)	0.09 ± 0.03 a	0.10 ± 0.03 b	0.59 ± 0.47 c	0.25 ± 0.04 d
P (mg/L)	0.25 ± 0.50 a	0.75 ± 0.50 a	0.00 ± 0.00 a	0.00 ± 0.00 a
K (mg/L)	393.50 ± 120.33 a	427.50 ± 57.93 a	216.75 ± 48.40 b	184.50 ± 27.72 c
Ca (mg/L)	1879.50 ± 587.38 a	2237.75 ± 318.91 a	1493.50 ± 456.59 a	1108.50 ± 160.48 b
Mg (mg/L)	575.00 ± 262.32 a	622.25 ± 60.84 a	349.75 ± 159.70 a	330.25 ± 138.75 a
OC (%)	0.31 ± 0.42 a	0.10 ± 0.00 a	0.11 ± 0.0 a	0.11 ± 0.01 a
Sand (%)	65.00 ± 8.29 a	47.75 ± 23.60 b	72.00 ± 17.66 c	76.50 ± 3.00 d
Silt (%)	9.00 ± 2.94 a	19.75 ± 11.38 a	11.75 ± 12.87 a	10.25 ± 0.96 a
Clay (%)	26.00 ± 6.27 a	33.25 ± 13.52 a	16.25 ± 4.50 b	13.25 ± 3.20 c

Mean ± standard deviation (*n* = 4); means in the same row with different letters were significantly different (*p* < 0.05) based on Tukey’s pairwise significant difference test. T1 = termite mounds from Braklaagte; T2 = termite mounds from Zeerust; S1 = surrounding soils from Braklaagte; and S2 = surrounding soils from Zeerust.

**Table 2:** Diversity indices for archaeal communities from termite mounds and surrounding soils.

		T1	T2	S1	S2	*p*-Value
Taxonomic	Shannon_H	2.69 ± 0.17	2.67 ± 0.18	2.68 ± 0.16	2.66 ± 0.18	0.99
composition	Evenness_e^H/S	0.87 ± 0.07	0.85 ± 0.07	0.86 ± 0.06	0.84 ± 0.07	0.99
Functional	Shannon_H	2.88 ± 0.13	2.87 ± 0.01	2.79 ± 1.57	2.39 ± 1.18	0.20
category	Evenness_e^H/S	0.64 ± 0.05	0.61 ± 0.02	0.63 ± 0.37	0.40 ± 0.28	0.20

T1 = termite mounds from Braklaagte; T2 = termite mounds from Zeerust; S1 = surrounding soils from Braklaagte; and S2 = surrounding soils from Zeerust.

## Data Availability

The quality sequences are available from the NCBI SRA dataset under the bioproject PRJNA526912 for the termite mound soil samples and PRJNA525146 for the surrounding soil samples.

## Supplementary Materials

The following are available online at https://www.mdpi.com/2079-7737/9/6/136/s1: Table S1: soil properties in termite mound soils and surrounding soils; Table S2: abundance of the archaeal communities at the phylum level; Table S3: abundance of the archaeal communities at the family level; Table S4: abundance of the archaeal communities at the genus level; Table S5: the abundance of the major functional categories in each soil sample; Figure S1: principal component analysis (PCA), showing the archaeal distribution at each site at the genus level.
