# Supplementary material for: Termite Societies Promote the Taxonomic and Functional Diversity of Archaeal Communities in Mound Soils
Source: Biology (Basel). 2020 Jun 25;9(6):136. doi: 10.3390/biology9060136 (PMC7345372; doi:10.3390/biology9060136)
Supplement: Supplementary file 1 [file biology-09-00136-s001.pdf]

Termite Societies Promote the Taxonomic and Functional Diversity of Archaeal Communities in Mound Soils

Monde Wakung'oli, Adenike Eunice Amoo, Ben Jesuorsemwen Enagbonma, and Olubukola Oluranti Babalola*

Supplementary

Table S1: Soil properties in termite mound soils and surrounding soils

Site	T1	T1	T1	T1	T2	T2	T2	T2	S1	S1	S1	S1	S2	S2	S2	S2
Sand	71	70	66	53	65	25	30	71	80	46	80	82	75	79	73	79
Silt	8	9	6	13	12	29	30	8	6	31	6	4	9	10	11	11
Clay	21	21	28	34	23	49	40	21	14	23	14	14	16	11	16	10
K	507	304	276	487	403	459	489	359	186	285	217	179	208	209	160	161
Ca	1500	1278	2215	2525	1790	2237	2417	2507	2010	997	1046	1921	873	1211	1140	1210
Mg	253	503	672	872	632	535	647	675	343	560	324	172	414	427	128	352
pH	4.89	4.79	5.52	5.21	4.21	4.52	5.11	4.06	5.64	5.4	5.97	6.14	5.84	5.57	5.09	5.03
N	0.13	0.07	0.07	0.1	0.14	0.09	0.1	0.07	1	0.1	0.26	1	0.29	0.22	0.22	0.27
P	1	0	0	0	1	0	1	1	0	0	0	0	0	0	0	0
OC	0.1	0.1	0.1	0.94	0.1	0.1	0.1	0.1	0.1	0.1	0.12	0.11	0.1	0.11	0.1	0.11

Table S2: Abundance of the archaeal communities at the phylum level

Phylum	T1	T1	T1	T1	T2	T2	T2	T2	S1	S1	S1	S1	S2	S2	S2	S2
Crenarchaeota	2146	2143	1553	1388	563	825	645	636	2478	1708	1324	1632	1448	1492	1381	2182
Euryarchaeota	7140	6696	6137	4463	1599	3270	1576	1936	5366	5820	5787	5384	5284	5127	3996	5679
Korarchaeota	99	86	62	41	16	37	31	22	60	43	67	52	47	49	37	63
Nanoarchaeota	7	5	3	2	2	0	1	0	1	0	3	2	2	1	2	0
Thaumarchaeota	3830	4173	2874	3013	1149	1269	1207	1449	5602	3508	1782	3004	2546	3066	2575	5577
unclassified Archaea	462	392	292	250	78	152	112	108	246	276	305	296	274	280	232	235

Table S3: Abundance of the archaeal communities at the family level

Family	T1	T1	T1	T1	T2	T2	T2	T2	S1	S1	S1	S1	S2	S2	S2	S2
Halobacteriaceae	2225	2239	2074	1550	488	1124	486	686	2004	2176	2000	1863	1879	1783	1465	2106
Methanosarcinaceae	825	776	610	622	205	433	212	221	667	704	554	754	685	630	536	635
Nitrosopumilaceae	629	722	495	508	176	231	190	274	973	568	313	484	438	525	432	955
Thermococcaceae	533	487	472	294	116	223	130	154	309	394	416	338	346	336	257	352
unclassified (derived from Archaea)	462	392	292	250	78	152	112	108	246	276	305	296	274	280	232	235
Cenarchaeaceae	378	423	296	293	130	160	144	132	458	333	192	252	218	238	246	453
Sulfolobaceae	419	347	300	211	89	142	97	78	340	280	247	206	245	176	221	311
Thermoproteaceae	366	362	280	235	80	165	107	95	270	255	296	242	234	195	215	280
Archaeoglobaceae	311	222	212	154	60	98	64	56	150	155	189	175	164	168	108	194
Desulfurococcaceae	248	278	191	173	64	125	52	77	238	204	212	196	196	182	172	199

Methanobacteriaceae	223	206	187	154	44	87	58	72	165	184	162	169	169	174	100	176
Methanomicrobiaceae	220	201	171	146	52	119	65	105	153	201	159	192	176	168	137	166
Methanocellaceae	237	171	190	124	56	66	30	49	150	169	155	161	178	152	139	144
Methanosaetaceae	116	119	87	75	56	66	30	49	63	93	110	89	62	56	61	87
Methanocaldococcaceae	113	107	82	63	17	61	28	31	89	86	80	53	83	81	56	78
Methanospirillaceae	94	89	86	92	28	34	22	21	91	93	82	99	101	98	82	83

Table S4: Abundance of the archaeal communities at the genus level

Genus	**T1**	**T1**	**T1**	**T1**	**T2**	**T2**	**T2**	**T2**	**S1**	**S1**	**S1**	**S1**	**S2**	**S2**	**S2**	**S2**
Nitrosopumilus	629	722	495	508	176	231	190	274	973	568	313	484	438	525	432	955
Methanosarcina	622	580	454	490	151	322	158	171	506	554	406	577	514	462	422	489
Candidatus Nitrososphaera	468	575	395	410	199	152	161	169	743	458	249	385	301	396	373	756
unclassified Archaea	462	392	292	250	78	152	112	108	246	276	305	296	274	280	232	235
Cenarchaeum	378	423	296	293	130	160	144	132	458	333	192	252	218	238	246	453
Thermococcus	304	273	263	180	51	128	74	87	183	232	234	190	200	191	139	218
Sulfolobus	306	266	219	159	71	112	82	59	240	212	190	151	184	143	186	238
Haloterrigena	235	259	230	189	46	111	49	95	232	262	209	220	239	224	175	220
Methanosphaera	22	20	14	16	6	8	1	5	20	32	15	20	23	20	20	18
Haladaptatus	198	205	225	137	52	118	30	61	219	221	199	175	184	144	156	207
Halalkalicoccus	208	216	185	133	57	106	44	51	191	207	171	207	151	161	146	189
Methanocella	237	171	190	124	56	66	30	49	150	169	155	161	178	152	139	144
Haloarcula	169	141	125	88	38	65	32	49	131	137	147	121	114	77	93	150
Methanoculleus	152	133	113	107	31	53	44	37	114	143	103	137	117	122	103	113
Archaeoglobus	161	121	129	92	32	59	32	39	97	100	114	110	117	95	61	120
Haloferax	136	131	129	101	38	80	29	40	131	136	115	111	113	105	107	119
Halogeometricum	138	137	106	95	22	50	40	40	105	124	108	102	106	80	72	114

Table S5: The abundance of the major functional categories in each soil sample

**functional categories**	**T1**	**T1**	**T1**	**T1**	**T2**	**T2**	**T2**	**T2**	**S1**	**S1**	**S1**	**S1**	**S2**	**S2**	**S2**	**S2**
Carbohydrates	88	111	16	773	1142	3118	984	963	82	0	70	0	355	28	255	199
Amino Acids and Derivaties	65	113	14	560	981	2464	739	772	42	0	42	3	237	6	143	149
Nitrogen Metabolism	9	9	0	61	79	239	50	40	4	0	5	0	24	2	5	10
Phosphorous Metabolism	8	8	1	120	175	453	147	147	10	0	7	0	27	0	20	16
Sulfur Metabolism	3	3	0	57	66	154	62	39	4	0	4	0	15	2	9	14
Potassium Metabolism	0	8	1	28	38	89	21	20	0	0	0	0	7	1	2	2

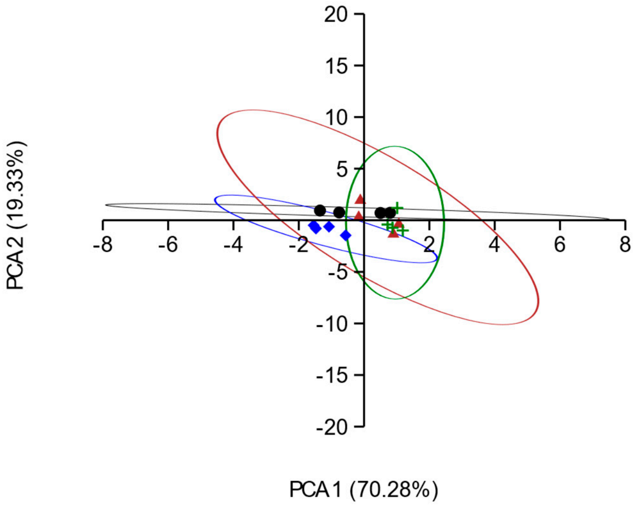

Figure S1: Principal component analysis (PCA), showing the archaeal distribution at each site at the genus level.
